# Bushels of Pills

**Published:** 1858-04

**Authors:** 


### BUSHELS OF PILLS.

A RELIGIOUS newspaper of this city repeats a reported saying, that several thousands of bushels of pills are swallowed every year, exclaiming:

"THIS IS CERTAINLY BAD!"

In another part of that same paper, an advertisement declares that a certain article is the "best and cheapest preparation for the hair ever made," that "anybody who uses it will be convinced of the above fact," and that "nobody will doubt its excellence after once using it, and no one desiring a fine head of hair should fail to use it."

We believe that it would require considerable ingenuity to incorporate as many demonstrable falsehoods in as few words. The best preparation for a fine head of hair is good health; the best and cheapest means are the proper use of a hair-brush and pure water.

In the same paper it is stated, that somebody's pills and powders "purify the blood, vitalize the lungs, impart energy to the nervous system, invigorate the liver, and increase digestion," and yet, under an editorial article commending "Ingenuousness," and ridiculing "Quack Medicines," the inquiry is made: "Are there no shams talked up, preached up, and also prayed up?" It might have been inquired if there were no shams printed in that same paper—for pay—for the Dollar?

Come out from the world, gentlemen! let the line of demarkation between you and secular editors be so distinct, that all may see it without the use of a microscope; otherwise, your doctrines and advertisements may be considered pretty much of the same sort—a humbug and a sham!

